# Synthesis of Novel UV Absorbers Bisindolylmethanes and Investigation of Their Applications on Cotton-Based Textile Materials

**DOI:** 10.3390/molecules21060718

**Published:** 2016-06-03

**Authors:** Hikmet Nil Ergindemir, Acelya Aker, Agamirze Hamitbeyli, Nuket Ocal

**Affiliations:** 1 Sanko Holding, ISKO Division, Organize Sanayi Bölgesi, 3. Cadde, İnegöl, Bursa 16400, Turkey; nergindemir@isko.com.tr (H.N.E.); mgamidov@isko.com.tr (A.H.); 2 Department of Chemistry, Faculty of Art and Sciences, Yildiz Technical University, Davutpasa Campus, Esenler, Istanbul 34220, Turkey; akeracelya@hotmail.com

**Keywords:** UV protection, indolyl compounds, textile applications

## Abstract

Nowadays modified textiles, especially UV-protective, antibacterial and antimicrobial ones, have become the focus of great interest. In this study, several new UV absorbers, bis(indolyl)methane derivatives, were synthesized and grafted onto polyvinyl alcohol polymer (PVA). Their application properties on cotton-based textile materials were determined; the UV protection factor values of the modified fabrics were measured (UPF); and the antibacterial features of the fabrics were tested.

## 1. Introduction

As a consequence of ozone layer deterioration, more UV radiation reaches the Earth’s surface, a major part of which is destructive to human health. It is known that long-term exposure to UV radiation causes harmful effects on the human skin, some of which are sunburn, erythema, skin aging and, most importantly, the risk of skin cancer [1,2]. In this case, the use of protective textile garments becomes a focus of great interest. UV radiation also causes unfavorable effects on synthetic materials. In wearable textiles, changes due to UV radiation can be observed in the physical and mechanical properties of synthetic materials such as color fading or decrease in tensile strength [3]. Previously, some studies proved that dye on fabrics reduces the transmission of ultraviolet radiation through the fabrics [4,5,6,7,8,9,10], but the light-induced fading of dyes is another problem. Nevertheless, light, bleached, uncolored cotton fabrics, which are especially needed in summer, usually do not provide enough UV protection. Protection properties of such textiles should be improved. Modifying cotton with light-absorbing materials in the UV range, especially in the UVB and UVA ranges, can be a solution for this problem. For effective application on cotton- or cellulose-based materials, UV absorbers should contain reactive groups to form covalent bonds with cellulose as well as UV-absorbing parts to increase the protection properties. A number of different types of UV absorbers have been proposed, but mostly benzophenone, phenyl ester, cinnamic acid and hydroxyphenylbenzotriazole derivatives could be of commercial value [11]. Inorganic salts can be used for modification of cotton fabrics with UV absorbers, but removal of solid waste products is problematic and causes environmental damage. Most of the formerly researched UV absorbers are water-insoluble and applications of dispersions and suspensions on fabrics are difficult [12,13,14]. Furthermore, these types of noncovalent interactions between cotton and organic molecules are weak and it is hard to keep the molecules on the cotton’s surface. Previously, some water-soluble and sulphonic acid groups containing UV absorbers were synthesized and applied to cotton [15,16,17,18], but a wide range of applications could not be found. There are some studies in the literature where brominated benzotriazole-type UV absorbers were grafted onto polyvinyl amine and applied on cotton [19]. In a similar study, an application was made using an electrostatic layer-by-layer self-assembly technique and increased washing fastness was observed [20].

Heterocyclic compounds are found in many natural and synthetic products with a wide range of pharmacological activities, such as antiviral, anticancer, antibacterial, antifungal, anticonvulsant, antiparkinsonian and anti-inflammatory activities, which can be well illustrated by the large number of drugs in the market containing this function group. We know that the reason for the very important biological activities of this type of heterocyclic organic compound is because of the presence of its characteristic N-C-O groups. The indole ring system is the most important heterocyclic group available in natural compounds. Several indole derivatives are reported to have antimicrobial activity [21]. It is known that bis(indolyl)methanes induce apoptosis in human cancer cells [22], and their derivatives are known as important intermediates in organic synthesis and pharmaceutical chemistry and they exhibit various physiological properties. Previously, the reactions of indole with carbonyl groups were studied in the presence of catalytic amounts of 1,3-dibromo-5,5-dimethylhydantoin and the corresponding bis(indolyl)methanes were obtained [23]. In this study, several UV-active bis(indolyl)methanes were prepared as new compounds and the UV-absorbing behaviors were investigated both in solution and polymer substrate. Polymers were dissolved and printed on 100% cotton undyed fabrics. UPF (Ultraviolet Protection Factor) values of the fabrics were measured and antibacterial effects were tested. In the textile field, these compounds that showed good UV absorber properties have been examined by us for the first time. 

## 2. Results and Discussion

### 2.1. Synthesis

**Scheme 1:**
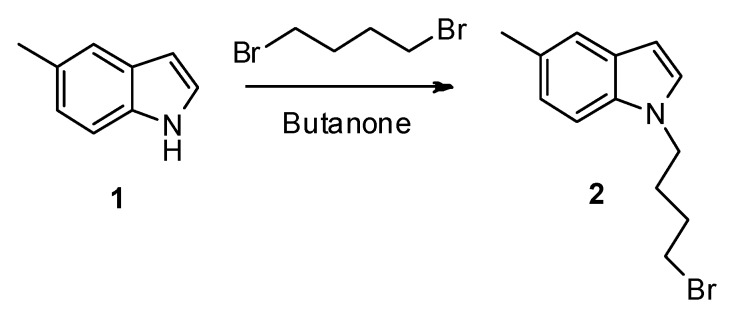
Preparation of **2**.

**Scheme 2:**
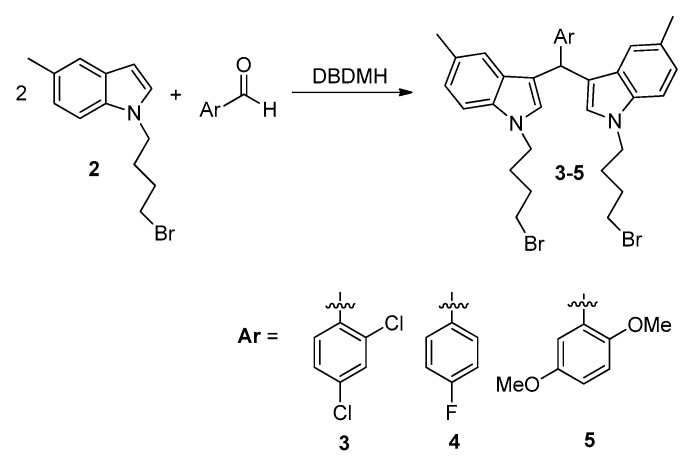
Synthesis of **3**–**5**.

Our synthesis started with the reaction of 5-methyl-1*H*-indole (**1**) with 1,4-dibromobutane to afford 1-(4-bromobutyl)-5-methyl-1*H*-indole (**2**) as the new compound which was reacted with 2,4-dichlorobenzaldehyde, *p*-fluorobenzaldehyde and 2,5-dimethoxybenzaldehyde in the presence of 1,3-dibromo-5,5-dimethylhydantoin (DBDMH) to give **3**–**5** in good yields (Scheme 1 and Scheme 2).

It is easy to see compounds **3**–**5** as UV-absorbing chromophores from their UV spectra (Table 1). The ^1^H-NMR spectra of **3**–**5** displayed singlets at 5.78–6.13 ppm due to CH protons in addition to other signals. In the IR spectra of the compounds, carbonyl bands were absent which clearly confirmed that a condensation with aryl aldehydes had taken place. In addition to the ^13^C-NMR and FTIR spectral data which were in agreement with the proposed structures, the mass spectra of all new compounds showed the expected molecular ion peaks.

**Scheme 3:**
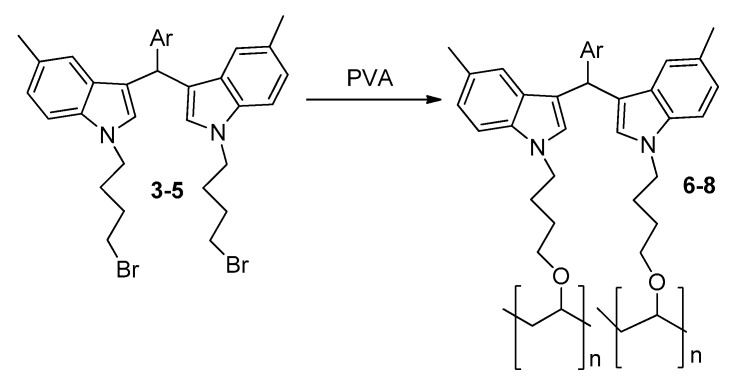
Polymers **6**–**8**.

Compounds **3**–**5** were grafted with PVA in DMF as a solvent to give polymers **6**–**8** for textile applications (Scheme 3).

**Scheme 4:**
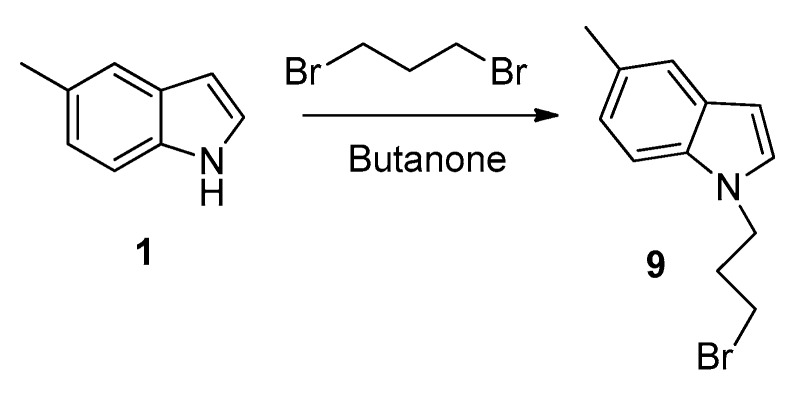
Preparation of **9**.

**Scheme 5:**
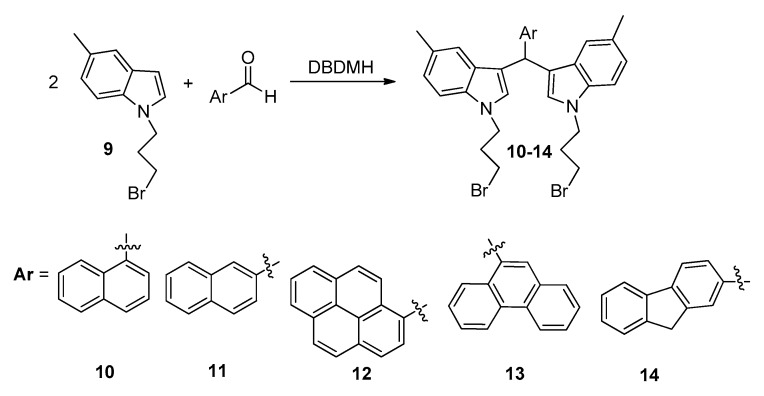
Synthesis of **10**–**14**.

**Scheme 6:**
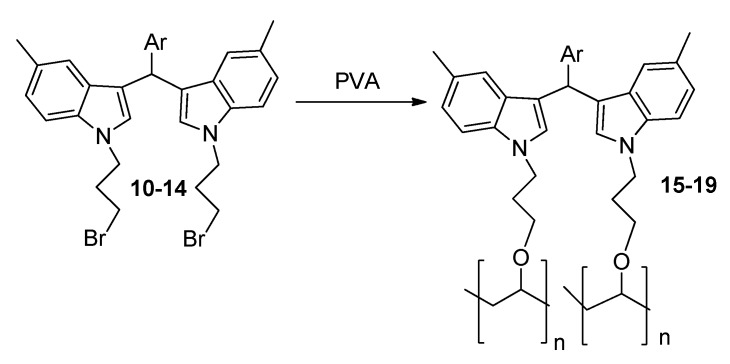
Synthesis of **15**–**19**.

We also synthesized compounds **10**–**14** as new compounds from **9** which were prepared from **1** with 1,3-dibromopropane with 1-naphtaldehyde, 2-naphtaldehyde, pyrene-1-carbaldehyde, phenanthrene-9-carbaldehyde and 9*H*-fluorene-2-carbaldehyde, respectively, under the same conditions (Scheme 4 and Scheme 5). Introduction of these aldehydes into the indolyl group chromophore improved their UV absorbance. The configuration of the new compounds was inferred from their NMR, IR and mass data. The ^1^H-NMR spectra of compounds **10**–**14** contained the singlet peaks of CH beetween two indole rings, indicating that the reaction took place. Compounds **10**–**14** were also grafted with PVA to give **15**–**19** for textile applications (Scheme 6).

**Figure 1:**
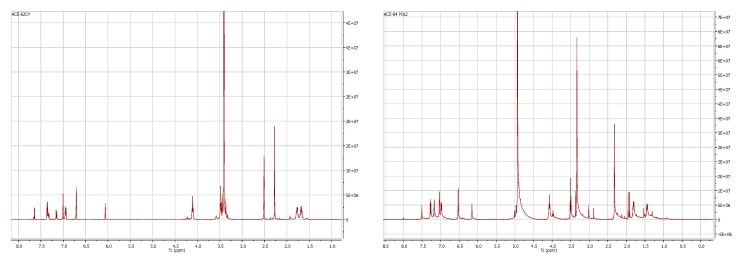
^1^H-NMR spectra of compounds **3** and **6**, respectively, in DMSO-*d*_6_.

**Figure 2:**
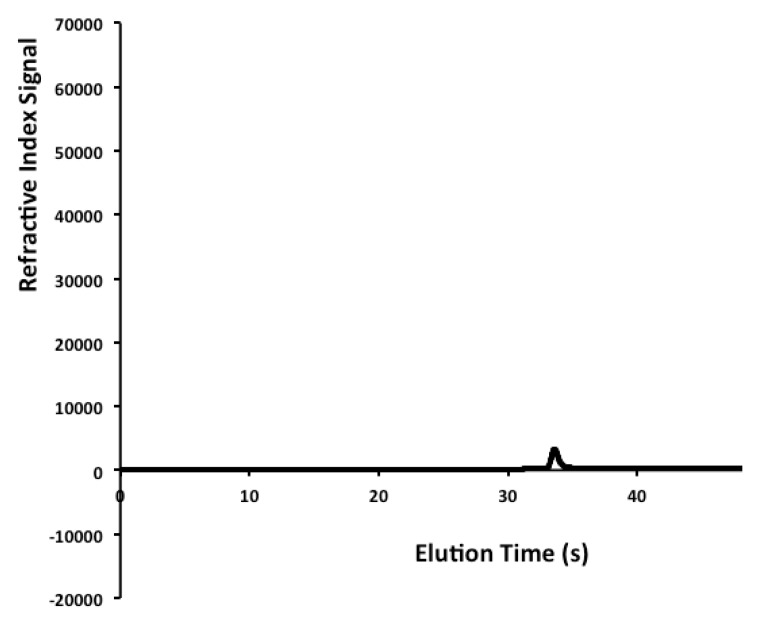
GPC result of compound **3**.

The new polymers (**6**–**8**; **15**–**19**) were characterized by ^1^H-NMR and GPC. The ^1^H-NMR spectra of all polymeric compounds were taken in DMSO-*d*_6_ and compared with response compounds. The molecular weights (M_w_ and M_n_) of the polymers were measured by GPC and summarized in Table 2. Figure 1 shows ^1^H-NMR spectra of compound **3** and its polymer **6** and Figure 2 also shows the GPC result of compound **3** as an example.

### 2.2. Textile Applications

Compounds **6**–**8** and **15**–**19** were dissolved in DMSO, mixed with printing paste and applied on 100% cotton undyed fabrics, using a flatbed screen method [24]. The fabrics were tested during AATCC 183:2014 standard at a temperature of 21 °C and 66% relative humidity [25]. The results showed that the UPF value of 27 of standard fabric increases up to 40+. According to standard specimens a UPF, UV-protection value between 15 and 24 is good, between 25 and 39 is very good, and 40 or greater is excellent. That means both UVA and UVB transmission through the fabrics decreased and excellent protection was obtained (Table 3). All printed fabrics were washed 25 times according to the BS EN ISO 6330 5A standard [26]. The fabrics’ UPF values were also tested after 25 washing cycles. The UPF values are the same as the unwashed fabrics, at 40+. In accordance with this, we still have the protective property after multiple washing cycles.

Antibacterial activities were tested according to the AATCC147-2011 standard, at a temperature of 37 ± 2 °C, and 18–24 h incubation time for the *Staphylococcus aureus* (AATCC 6538 Gram+) microorganism [27]. The results showed that antibacterial activities were achieved (Table 4). We used nontoxic materials at both synthesis and application steps. Nevertheless, we tested the coated fabrics during the OEKO TEX 100 standard for PAH (Polyaromatic hydrocarbon), APEO (Alkylphenyl etoxylate) and phthalate contents. According to the results, it can be said that the fabrics do not have toxic effects.

## 3. Materials and Methods

### 3.1. General Information

Reactions were monitored using thin-layer chromatography (TLC). Resulting residue was purified by silica gel chromatography with a solvent mixture to afford the title compounds. PVA was used as Mowiol 1888: Ma: 130,000 g/mol, Pw: 2700 was supplied from Kuraray. UV absorption spectra were recorded on an Agilent Technologies 8453 spectrophotometer (Santa Clara, CA, USA). IR spectra were obtained with a “Perkin Elmer, FT-IR” system and were reported in terms of frequency of absorption (cm^−1^). Melting points were determined with “Gallenkamp” digital thermometer equipment (Northhampton, UK). All melting points are uncorrected. NMR spectra were determined with a “Bruker Ac-500 MHz NMR” (Rheinstetten, Germany). TMS (tetramethylsilane) was used as the internal standard and CDCl_3_ was used as the solvent. Signal multiplicities in the NMR spectra were reported as follows: s—singlet, brs—broad singlet, d—doublet, dd—doublet of doublets, and m—multiplet. Mass spectra were measured with an Agilent 6890N GC-System-5973 IMSD or Agilent LC-MSD Trap SL. Gel Permeation Chromatography (Visotek) was carried out with THF solvent using polystyrene standards. All tests related to textile applications were done by third party laboratories having accreditation. Transmittance or blocking tests of erythemally weighted ultraviolet radiation through fabrics (AATCC 183:2014) and toxicity tests (EOKOTEX 100) were conducted in Intertek laboratories, Istanbul/Turkey. Domestic washing and drying procedures tests for textiles (BS EN ISO 6330 5A) were done by Sanko Holding-ISKO division, R & D laboratories, Bursa/Turkey. Antibacterial activity assessments of textile materials (AATCC147-2011) tests were conducted in Ekoteks laboratories, Istanbul/Turkey.

### 3.2. Synthesis of ***2*** and ***9***

To 5-methyl-1*H*-indole (2 mmol) dissolved in 5 mL 2-butanone at 55 °C, KOH (4 mmol) was added and the mixture stirred for 1 h. Then 1,4-dibromobutane for compound (**2**) and 1,3-dibromopropane for compound (**9**) was added and the reaction completed in 2 h. The progress of the reaction was monitored by TLC. The residue was removed and the mixture evaporated and purified by column chromatography (10:1; *n*-hexane/EtOAc).

*1-(4-Bromobutyl)-5-methyl-1H-indole* (**2**). Colorless oil; Yield = 70%; R_f_ = 0.76 (1:5; EtOAc/*n-*hexane); FTIR (ATR): υ = 3032, 3011, 2918, 2851, 1673, 1488, 1445, 1355 cm^−1^; ^1^H-NMR (CDCl_3_, 500 MHz): δ = 1.72 (p, *J* = 6.62 Hz, 2H, CH_2_), 1.88 (p, *J* = 6.9 Hz, 2H, CH_2_), 2.36 (s, 3H, CH_3_), 3.25 (t, *J* = 6.62 Hz, 2H, CH_2_), 4.02 (t, *J* = 6.62 Hz, 2H, CH_2_), 6.32 (d, *J* = 3.15 Hz, 1H, ArH), 6.94 (d, *J* = 3.15 Hz, 2H, ArH), 7.13 (d, *J* = 8.51 Hz, 1H, ArH), 7.33 (s, 1H, ArH) ppm; GC-MS (EI, 70 eV): *m*/*z* = 265 (M^+^).

*1-(3-Bromopropyl)-5-methyl-1H-indole* (**9**). Colorless oil; Yield = 75%; R_f_ = 0.82 (1:10; EtOAc/*n-*hexane); FTIR (ATR): υ = 3023, 3001, 2942, 2853, 1670, 1501, 1443, 1355 cm^−1^; ^1^H-NMR (CDCl_3_, 500 MHz): δ = 2.24 (p, *J* = 6.30 Hz, 2H, CH_2_), 2.37 (s, 3H, CH_3_), 3.20 (t, *J* = 6.30 Hz, 2H, CH_2_), 4.21 (t, *J* = 6.30 Hz, 2H, CH_2_), 6.33 (dd, *J* = 0.94; 3.15 Hz, 1H, =CH), 6.96 (dd, *J* =1.57; 8.51 Hz, 1H, =CH), 7.01 (d, *J* = 3.15 Hz, 1H, ArH), 7.18 (d, *J* = 8.19 Hz, 1H, ArH), 7.34 (s, 1H, ArH) ppm; LCMS (ESI-QTOF) *m*/*z*: calcd for C_12_H_14_BrN 251.0309; Found 253.0361 [M + H]^+^.

### 3.3. General Procedure of Bromoalkylbis(indolyl)methanes *(**3***–***5***, ***10***–***14**)*

The syntheses were accomplished according to the general procedure in the literature [23]. A mixture of carbonyl compound/indole/DBDMH: 2/1/0.05 mmol was stirred at 55 °C under solvent-free conditions for 2 h. The progress of the reaction was monitored by TLC (15:1; *n*-hexane/EtOAc). The residue recrystallized from methanol.

*3,3′-((2,4-Dichlorophenyl)methylene)bis(1-(4-bromobutyl)-5-methyl-1H-indole* (**3**). Colorless crystals; mp 141–142 °C; Yield = 55%; R_f_ = 0.32 (1:8; EtOAc/*n-*hexane);FTIR (ATR): υ = 3081, 3012, 2927, 2874, 2856, 1585, 1463, 1359, 1100 cm^−1^; ^1^H-NMR (DMSO-*d*_6_, 500 MHz): δ = 1.64–1.80 (m, 8H, CH_2_), 2.27 (s, 6H, CH_3_), 3.47 (t, *J* = 6.62 Hz, 4H, CH_2_), 4.10 (t, *J* = 6.62 Hz, 4H, CH_2_), 6.05 (s, 1H, CH), 6.70 (s, 2H, ArH), 6.94 (d, *J* = 8.19 Hz, 2H, ArH), 7.00 (s, 2H, ArH), 7.15 (d, *J* = 8.51 Hz, 1H, ArH), 7.31 (dd, *J* = 2.20; 8.51 Hz, 1H, ArH), 7.35 (d, *J* = 8.19 Hz, 2H, ArH), 7.63 (s, 1H, ArH) ppm; ^13^C-NMR (DMSO-*d*_6_, 125 MHz): δ = 21.1 (2 × CH_3_), 28.4 (2 × CH_2_), 34.5 (2 × CH_2_), 35.6 (CH), 44.4 (CH_2_), 109.7 (=CH), 114.7 (Cq), 118.3 (2 × CAr), 122.8 (2 × CAr), 126.9 (Cq), 127.1 (Cq), 127.2 (CAr), 128.7 (CAr), 131.1 (CAr), 133.6 (Cq), 134.7 (Cq), 140.8 (Cq) ppm; UV (λ_max_, CH_2_Cl_2_): 315 nm (*c* = 3.5 × 10^−4^, A = 1.52, ε = 4 × 10^−3^); LCMS (ESI-QTOF) *m*/*z:* calcd for C_33_H_34_Br_2_Cl_2_N_2_ 686.0465; Found 687.0522 [M + H]^+^.

*3,3′-((4-Fluorophenyl)methylene)bis(1-(4-bromobutyl)-5-methyl-1H-indole* (**4**). Colorless crystals; mp 118–119 °C; Yield = 67%; R_f_ = 0.22 (1:8; EtOAc/*n-*hexane); FTIR (ATR**):** υ = 3028, 3009, 2957, 2920, 2854, 1602, 1549, 1504, 1485, 1453, 1359, 1012 cm^−1^; ^1^H-NMR (DMSO-*d*_6_, 500 MHz): δ = 1.67–1.71 (p, 4H, CH_2_), 1.75–1.80 (p, 4H, CH_2_), 2.27 (s, 6H, CH_3_), 3.48 (t, *J* = 6.62 Hz, 4H, CH_2_), 4.10 (t, *J* = 6.62 Hz, 4H, CH_2_), 5.78 (s, 1H, CH), 6.75 (s, 2H, ArH), 6.92 (d, *J* = 8.19 Hz, 2H, ArH), 7.06 (s, 2H, ArH), 7.10 (d, *J* = 8.82 Hz, 2H, ArH), 7.31–7.34 (m, 4H, ArH) ppm; ^13^C-NMR (CDCl_3_, 125 MHz): δ = 21.5 (2 × CH_3_), 28.8 (2 × CH_2_), 30.0 (2 × CH_2_), 32.1 (CH), 33.1 (2 × CH_2_), 45.3 (2 × CH_2_), 55.5 (CH_3_), 56.6 (CH_3_), 108.8 (CAr), 110.6 (CAr), 111.8 (CAr), 116.5 CAr), 117.6 (Cq), 119.9 (CAr), 122.9 (CAr), 127.2 (CAr), 127.7 (Cq), 128.0 (Cq), 134.3 (Cq), 135.0 (Cq), 151.4 (Cq), 153.4 (Cq) ppm; UV (λ_max_, CH_2_Cl_2_**)**: 310 nm (*c* = 2.2 × 10^−4^, A = 1.42, ε = 6.4 × 10^3^); LCMS (ESI-QTOF) *m*/*z:* calcd for C_33_H_35_Br_2_FN_2_ 636.1150; Found 637.1241 [M + H]^+^.

*3,3′-((2,5-Dimethoxyphenyl)methylene)bis(1-(4-bromobutyl)-5-methyl-1H-indole* (**5**). Colorless crystals; mp 102 °C; Yield = 60%; R_f_ = 0.12 (1:15; EtOAc/*n-*hexane); FTIR (ATR): υ = 3022, 3000, 2939, 2862, 2831, 1616, 1588, 1545, 1489, 1450, 1363, 1024 cm^−1^; ^1^H-NMR (DMSO-*d*_6_, 500 MHz): δ = 1.72–1.76 (p, 4H, CH_2_), 1.80–1.85 (p, 4H, CH_2_), 2.32 (s, 6H, CH_3_), 3.53 (t, *J* = 6.62 Hz, 4H, CH_2_), 3.62 (s, 3H, OCH_3_), 3.79 (s, 3H, OCH_3_), 4.15 (t, *J* = 6.62 Hz, 4H, CH_2_), 6.13 (s, 1H, CH), 6.66 (d, *J* = 2.83 Hz, 1H, ArH), 6.76 (s, 2H, ArH), 6.80 (dd, *J* = 3.15; 8.82 Hz, 1H, ArH), 6.97 (d, *J* = 8.51 Hz, 2H, ArH), 7.01 (d, *J* = 8.82 Hz, 1H, ArH), 7.07 (s, 2H, ArH), 7.37 (d, *J* = 8.51 Hz, 2H, ArH) ppm; ^13^C-NMR (CDCl_3_, 125 MHz): δ = 21.5 (2 × CH_3_), 28.8 (2 × CH_2_), 30.0 (2 × CH_2_), 32.1 (CH), 33.1 (2 × CH_2_), 45.3 (2 × CH_2_), 55.5 (CH_3_), 56.6 (CH_3_), 108.8 (CAr), 110.6 (CAr), 111.8 (CAr), 116.5 CAr), 117.6 (Cq), 119.9 (CAr), 122.9 (CAr), 127.2 (CAr), 127.7 (Cq), 128.0 (Cq), 134.3 (Cq), 135.0 (Cq), 151.4 (Cq), 153.4 (Cq) ppm; UV (λ_max_, CH_2_Cl_2_): 300 nm (*c* = 2.4 × 10^−4^, A = 1.77, ε = 7.2 × 10^3^); LCMS (ESI-QTOF) *m*/*z*: calcd for C_33_H_38_Br_2_N_2_O_2_ 677.1377; Found 678.1409 [M + H]^+^.

*3,3′-((Naphthalene-1-yl)methylene)bis(1-(4-bromopropyl)-5-methyl-1H-indole* (**10**). Colorless crystals; mp 127–130 °C; Yield = 61%; R_f_ = 0.58 (1:15; EtOAc/*n-*hexane); FTIR (ATR): υ = 3030, 3011, 2957, 2861, 1610, 1600, 1504, 1462, 1453, 1354, 1100 cm^−1^; ^1^H-NMR (CDCl_3_, 500 MHz): δ 2.13 (p, *J* = 6.30 Hz, 4H, CH_2_), 2.26 (s, 6H, CH_3_), 3.08 (p, *J* = 6.30 Hz, 4H, CH_2_), 4.05 (t, *J* = 6.30 Hz, 4H, CH_2_), 6.36 (s, 2H, =CH), 6.47 (s, 1H, CH), 6.95 (dd, *J* = 0.94; 8.19 Hz, 2H, ArH), 7.10 (s, 2H, ArH), 7.14 (d, *J* = 7.25 Hz, 1H, ArH), 7.17 (d, *J* = 8.19 Hz, 2H, ArH), 7.23–7.29 (m, 2H, ArH), 7.34–7.38 (m, 1H, ArH), 7.67 (d, *J* = 8.19 Hz, 1H, ArH), 7.80 (d, *J* = 7.88 Hz, 1H, ArH), 8.03 (d, *J* = 8.51 Hz, 1H, ArH) ppm; ^13^C-NMR (CDCl_3_, 125 MHz): δ = 21.3 (2 × CH_3_), 30.3 (2 × CH_2_), 32.7(2 × CH_2_), 35.8 (CH), 43.8 (2 × CH_2_), 109.0 (2 × CAr), 117.6 (2 × Cq), 119.7 (2 × CAr), 123.2 (CAr), 124.5 (CAr), 125.2 (CAr), 125.5 (CAr), 125.6 (CAr), 125.9 (CAr), 126.9 (CAr), 127.9 (2 × Cq), 128.2 (2 × Cq), 128.3 (CAr), 128.6 (CAr), 131.8 (Cq), 134.0 (Cq), 135.0 (2 × Cq), 139.7 (Cq) ppm; UV (λ_max_, CH_2_Cl_2_**)**: 290 nm (*c* = 2.0 × 10^−4^, A = 1.79, ε = 8.9 × 10^3^); LCMS (ESI-QTOF) *m*/*z*: calcd for C_35_H_34_Br_2_N_2_ 640.1088; Found 641.0988 [M + H]^+^.

*3,3′-((Naphthalene-2-yl)methylene)bis(1-(4-bromopropyl)-5-methyl-1H-indole* (**11**). Colorless crystals; mp 101–103 °C; Yield = 65%; R_f_ = 0.55 (1:15; EtOAc/*n-*hexane); FTIR (ATR): υ = 3022, 2964, 2920, 1628, 1597, 1522, 1482, 1362, 1222, 1088, 823 cm^−1^; ^1^H-NMR (CDCl_3_, 500 MHz):δ = 2.18 (p, *J* = 6.30 Hz, 4H, CH_2_), 2.25 (s, 6H, CH_3_), 3.16 (t, *J* = 6.30 Hz, 4H, CH_2_-N), 4.10 (p, *J* = 6.30 Hz, 4H, CH_2_-Br), 5.89 (s, 1H, CH), 6.47 (s, 2H, =CH), 6.95 (dd, *J* = 1.57; 8.51 Hz, 2H, ArH), 7.12 (s, 2H, ArH), 7.17 (d, *J* = 8.51 Hz, 2H, ArH), 7.34–7.35 (m, 2H, ArH), 7.42 (dd, *J* = 8.51 Hz, 1H, ArH), 7.64–7.70 (m, 3H, ArH), 7.73–7.75 (m, 1H, ArH) ppm; ^13^C-NMR (CDCl_3_, 125 MHz): δ = 21.4 (2 × CH_3_), 30.4 (2 × CH_2_), 32.8 (2 × CH_2_), 40.1 (CH), 44.0 (2 × CH_2_), 109.3 (2 × CAr), 116.6 (Cq), 118.0 (2 × Cq), 119.8 (2 × CAr), 123.3 (2 × CAr), 125.2 (CAr), 125.6 (CAr), 126.6 (CAr), 127.6 (CAr), 127.7 (2 × CAr), 127.9 (CAr), 128.2 (2 × Cq), 132.3 (Cq), 133.6 (2 × Cq), 135.0 (2 × Cq), 141.8 (Cq) ppm; UV (λ_max_, CH_2_Cl_2_**)**: 310 nm (*c* = 1.2 × 10^−4^, A = 1.77, ε = 1.4 × 10^4^); LCMS (ESI-QTOF) *m*/*z*: calcd for C_35_H_34_Br_2_N_2_ 640.1088; Found 641.1889 [M + H]^+^.

*3,3′-((Pyren-1-yl)methylene)bis(1-(4-bromopropyl)-5-methyl-1H-indole* (**12**). Colorless crystals; mp 133–135 °C; Yield = 72%; R_f_ = 0.49 (1:15; EtOAc/*n-*hexane); FTIR (ATR): υ = 3025, 2922, 1636, 1592, 1550, 1451, 1361, 1222, 1158, 785 cm^−1^; ^1^H-NMR (CDCl_3_, 500 MHz): δ = 2.13 (p, *J* = 6.30 Hz, 4H, CH_2_), 2.22 (s, 6H, CH_3_), 3.10 (t, *J* = 6.30 Hz, 4H, CH_2_), 4.03–4.06 (m, 4H, CH_2_), 6.35 (s, 2H, =CH), 6.79 (s, 1H, CH), 6.96 (dd, *J* = 1.26; 8.19 Hz, 2H, ArH), 7.09 (s, 2H, ArH), 7.19 (d, *J* = 8.19 Hz, 2H, ArH), 7.72 (d, *J* = 7.88 Hz, 1H, ArH), 7.91 (d, *J* = 8.82 Hz, 2H, ArH), 7.95–7.97 (m, 3H, ArH), 8.06–8.10 (m, 2H, ArH); 8.33 (d, *J* = 9.46 Hz, 1H, ArH) ppm; ^13^C-NMR (CDCl_3_, 125 MHz): δ = 21.4 (2 × CH_3_), 30.4 (CH_2_), 30.6 (CH_2_), 32.8 (2 × CH_2_), 36.3 (CH), 43.9 (2 × CH_2_), 100.9 (CAr), 108.9 (CAr), 109.0 (CAr), 116.5 (Cq), 117.0 (Cq), 118.0 (2 × Cq), 119.8 (CAr), 120.7 (CAr), 123.2 (CAr), 123.4 (CAr), 123.9 (CAr), 124.8 (CAr), 124.9 (CAr), 125.0 (Cq), 125.2 (Cq), 125.8 (CAr), 126.5 (CAr), 126.7 (CAr), 127.2 (CAr), 127.6 (CAr), 127.9 (Cq), 128.1 (CAr), 128.3 (Cq), 128.4 (Cq), 128.7 (Cq), 130.0 (Cq), 130.9 (Cq), 131.4 (Cq), 135.1 (Cq), 137.9 (Cq) ppm; UV (λ_max_, CH_2_Cl_2_**)**: 346 nm (*c* = 1.6 × 10^−4^, A= 1.60, ε = 1.0 × 10^4^); LCMS (ESI-QTOF) *m*/*z*: calcd for C_41_H_36_Br_2_N_2_ 714.1245; Found 685.0317 [M + H − 2Me]^+^.

*3,3′-((Phenanthren-9-yl)methylene)bis(1-(4-bromopropyl)-5-methyl-1H-indole* (**13**). Colorless crystals; mp 168–171 °C; Yield = 75%; R_f_ = 0.58 (1:15; EtOAc/*n-*hexane); FTIR (ATR): υ = 3071, 3021, 2961, 2921, 1603,1550, 1484, 1364, 1228, 1018, 785 cm^−1^; ^1^H-NMR (CDCl_3_, 500 MHz): δ = 2.12–2.16 (m, 4H, CH_2_), 2.25 (s, 6H, CH_3_), 3.04–3.09 (m, 4H, CH_2_), 4.04–4.07 (m, 4H, CH_2_), 6.39 (s, 1H, CH), 6.42 (s, 2H, =CH), 6.49 (d, *J* = 8.19 Hz, 2H, ArH),6.94–6.98 (m, 2H, ArH), 7.12 (d, *J* = 8.19 Hz, 1H, ArH), 7.16 (s, 2H, ArH), 7.18–7.20 (m, 2H, ArH), 7.37–7.43 (m, 2H, ArH), 7.50–7.56 (m, 2H, ArH), 8.10 (t, *J* = 8.19 Hz, 1H, ArH), 8.69 (d, *J* = 8.19 Hz, 1H, ArH) ppm; ^13^C-NMR (CDCl_3_, 125 MHz): δ = 21.3 (2 × CH_3_), 30.3 (2 × CH_2_), 32.7 (2 × CH_2_), 35.9 (CH), 43.9 (2 × CH_2_), 109.0 (CAr), 109.3 (CAr), 116.4 (2 × Cq), 117.3 (Cq), 117.5 (Cq), 119.4 (CAr), 119.6 (CAr), 119.8 (CAr), 122.3 (CAr), 122.9 (CAr), 123.2 (CAr), 123.3 (CAr), 125.2 (CAr), 125.9 (CAr), 126.0 (CAr), 126.3 (CAr), 126.5 (CAr), 128.0 (Cq), 128.2 (Cq), 128.3 (Cq), 128.5 (CAr), 128.8 (CAr), 129.9 (Cq), 130.8 (CAr), 131.4 (Cq), 131.8 (Cq), 133.8 (CAr), 135.1 (Cq), 138.0 (Cq) ppm; UV (λ_max_, CH_2_Cl_2_**)**: 296 nm (*c* = 1.6 × 10^−4^, A = 1.47, ε = 9.1 × 10^3^); LCMS (ESI-QTOF) *m*/*z*: calcd for C_39_H_36_Br_2_N_2_ 690.1245; Found 691.4354 [M + H]^+^.

*3,3′-((9H-Fluoren-2-yl)methylene)bis(1-(4-bromopropyl)-5-methyl-1H-indole* (**14**). Colorless crystals; mp 193–196 °C; Yield = 60%; R_f_ = 0.42 (1:15; EtOAc/*n-*hexane); FTIR (ATR): υ = 3019, 2959, 2916, 1608, 1573,1483, 1395, 1361, 1226, 1023, 790, 737 cm^−1^; ^1^H-NMR (CDCl_3_, 500 MHz): δ = 2.19 (p, *J* = 6.30 Hz, 4H, CH_2_), 2.26 (s, 6H, CH_3_), 3.17 (t, *J* = 6.30 Hz, 4H, CH_2_), 3.76 (s, 2H, CH_2_), 4.09–4.12 (m, 4H, CH_2_), 6.43 (s, 1H, CH), 6.48 (s, 2H, =CH), 6.94 (d, *J* = 8.51 Hz, 2H, ArH), 7.12 (d, *J* = 3.48 Hz, 2H, ArH), 7.17 (d, *J* = 8.51 Hz, 2H, ArH), 7.26–7.29 (m, 2H, ArH), 7.41 (s, 1H, ArH), 7.43 (d, *J* = 7.56 Hz, 1H, ArH), 7.62 (dd, *J* = 1.57; 7.88 Hz, 1H, ArH), 7.67 (d, *J* = 7.56 Hz, 1H, ArH), 7.84 (s, 1H, ArH) ppm; ^13^C-NMR (CDCl_3_, 125 MHz): δ = 21.4 (2 × CH_3_), 30.4 (2 × CH_2_), 32.8 (2 × CH_2_), 36.9 (CH_2_), 40.1 (CH), 43.9 (2 × CH_2_), 108.9 (CAr), 109.2 (CAr), 116.6 (Cq), 118.2 (Cq), 118.3 (Cq), 119.8 (CAr), 120.1 (CAr), 121.1 (CAr), 123.1 (CAr), 123.2 (CAr), 125.0 (CAr), 125.2 (CAr), 126.2 (CAr), 126.6 (CAr), 127.2 (CAr), 127.4 (CAr), 127.9 (Cq), 128.0 (Cq), 128.2 (Cq), 128.4 (CAr), 133.8 (CAr), 135.0 (Cq), 135.3 (Cq), 139.8 (Cq), 141.8 (Cq), 143.1 (Cq), 143.3 (Cq), 143.4 (Cq) ppm; UV (λ_max_, CH_2_Cl_2_**)**: 307 nm (*c* = 2.0 × 10^−4^, A = 1.50, ε = 7.5 × 10^3^); LCMS (ESI-QTOF) *m*/*z*: calcd for C_38_H_36_Br_2_N_2_ 678.1245; Found 679.1143 [M + H]^+^.

### 3.4. Synthesis of Polymers *(**6***–***8***, ***15***–***19**)*

First 40 mg PVA dissolved in 2 mL DMF at 120 °C, then 50 mg K_2_CO_3_ was added and stirred for 1 h. At the end of this time, 100 mg bromoalkylbis(indolyl)methane/1 mL DMF was added dropwise to the reaction mixture. The reaction was completed in 3 h. Crushed ice was added to the mixture and centrifuged and the residue dried in an oven.

*Polymer* (**6**). Light orange solid; yield = 47%; FTIR (ATR): υ = 3348, 3026, 2930, 2874, 1557, 1486, 1367, 1165, 1049, 831 cm^−1^; ^1^H-NMR (DMSO-*d*_6_, 500 MHz): δ = 1.30–1.32 (m, n6H, CH_3_), 1.41–1.47 (m, 8H, CH_2_), 1.50–1.53 (m, n6H, CH_3_), 1.78–1.84 (m, n4H, CH_2_), 2.32 (s, 6H, CH_3_), 3.32–3.33 (m, n2H, CH), 3.50 (t, *J* = 6.30 Hz, 4H, CH_2_), 4.07 (t, *J* = 6.93 Hz, 4H, CH_2_), 6.15 (s, 1H, CH), 6.53 (s, 2H, ArH), 6.98 (d, *J* = 8.19 Hz, 2H, ArH), 7.03 (s, 2H, ArH), 7.17 (d, *J* = 4.72 Hz, 2H, ArH), 7.27 (d, *J* = 8.51 Hz, 2H, ArH), 7.50 (d, *J* = 1.57 Hz, 1H, ArH) ppm; UV (λ_max_, DMSO**)**:315 nm (*c* = 0.5 × 10^−4^, A = 0.60, ε = 1.2 × 10^4^).

*Polymer* (**7**). Light brown solid; yield = 52%; FTIR (ATR): υ = 3340, 3043, 2932, 2855, 1661,1600, 1504, 1485, 1363, 1217, 1091, 793 cm^−1^; ^1^H-NMR (DMSO-*d*_6_, 500 MHz): δ = 1.29–1.31 (m, n6H, CH_3_), 1.39–1.45 (m, 8H, CH_2_), 1.49–1.51 (m, n6H, CH_3_), 1.77–1.83 (m, n4H, CH_2_), 2.31 (s, 6H, CH_3_), 3.31–3.34 (m, n2H, CH), 3.52 (t, *J* = 6.30 Hz, 4H, CH_2_), 4.12 (t, *J* = 6.93 Hz, 4H, CH_2_), 6.00 (s, 1H, CH), 6.73 (s, 2H, ArH), 6.91 (d, *J* = 8.51 Hz, 2H, ArH), 7.16 (s, 2H, ArH), 7.12 (d, *J* = 8.82 Hz, 2H, ArH), 7.31–7.34 (m, 4H, ArH) ppm; UV (λ_max_, DMSO**)**: 310 nm (*c* = 0.5 × 10^−4^, A = 1.90, ε = 3.8 × 10^4^).

*Polymer* (**8**). Orange solid; yield = 47%; FTIR (ATR): υ = 3372, 3019, 2933, 2871, 1661,1589, 1547, 1460, 1366, 1211, 1094, 714 cm^−1^; ^1^H-NMR (DMSO-*d*_6_, 500 MHz): δ = 1.38–1.39 (m, n6H, CH_3_), 1.43–1.49 (m, 8H, CH_2_), 1.51–1.54 (m, n6H, CH_3_), 1.79–1.85 (m, n4H, CH_2_), 2.30 (s, 6H, CH_3_), 3.41–3.45 (m, n2H, CH), 3.61 (s, 3H, OCH_3_), 3.50 (t, *J* = 6.30 Hz, 4H, CH_2_), 3.81 (s, 3H, OCH_3_), 4.07 (t, *J* = 6.93 Hz, 4H, CH_2_), 6.15 (s, 1H, CH), 6.64 (d, *J* = 3.15 Hz, 1H, ArH), 6.79 (s, 2H, ArH), 6.82 (dd, *J* = 3.15; 8.82 Hz, 1H, ArH), 6.99 (d, *J* = 8.19 Hz, 2H, ArH), 7.04 (d, *J* = 8.82 Hz, 1H, ArH), 7.08 (s, 2H, ArH), 7.34 (d, *J* = 8.51 Hz, 2H, ArH) ppm; UV (λ_max_, DMSO**)**: 315 nm (*c* = 0.5 × 10^−4^, A = 1.63, ε = 3.26 × 10^4^).

*Polymer* (**15**). Light brown solid; yield = 65%; FTIR (ATR): υ = 3381, 3041, 2918, 2858, 1662, 1595, 1485, 1435, 1385, 1173, 1089, 786 cm^−1^; ^1^H-NMR (DMSO-*d*_6_, 500 MHz): δ = 1.26–1.28 (m, n6H, CH_3_), 1.32–1.33 (m, n6H, CH_3_), 1.44–1.48 (m, 4H, CH_2_), 1.91–1.95 (m, 4H, CH_2_), 2.34 (s, 6H, CH_3_), 3.32–3.33 (m, n2H, CH), 3.37 (t, *J* = 6.30 Hz, 4H, CH_2_), 4.16 (t, *J* = 6.93 Hz, 4H, CH_2_), 6.26 (s, 2H, =CH), 6.47 (s, 1H, CH), 6.95 (dd, *J* = 0.94; 8.51 Hz, 2H, ArH), 7.09 (s, 2H, ArH), 7.12 (d, *J* = 7.25 Hz, 1H, ArH), 7.19 (d, *J* = 8.51 Hz, 2H, ArH), 7.22–7.31 (m, 2H,ArH), 7.34–7.38 (m, 1H, ArH), 7.66 (d, *J* = 8.51 Hz, 1H, ArH), 7.81 (d, *J* = 7.88 Hz, 1H, ArH), 8.12(d, *J* = 8.51 Hz, 1H, ArH) ppm; UV (λ_max_, DMSO**)**: 283 nm (*c* = 2.0 × 10^−4^, A = 1.20, ε = 6.0 × 10^3^).

*Polymer* (**16**). Light brown solid; yield = 60%; FTIR (ATR): υ = 3338, 3049, 2921, 2856, 1655, 1596, 1484, 1458, 1383, 1172, 1089, 788 cm^−1^; ^1^H-NMR (DMSO-*d*_6_, 500 MHz): δ = 1.18–1.21 (m, n6H, CH_3_), 1.29–1.31 (m, n6H, CH_3_), 1.41–1.45 (m, 4H, CH_2_), 1.89–1.93 (m, 4H, CH_2_), 2.31 (s, 6H, CH_3_), 3.31–3.34 (m, n2H, CH), 3.31 (t, *J* = 6.30 Hz, 4H, CH_2_), 4.06 (t, *J* = 6.93 Hz, 4H, CH_2_), 5.91 (s, 1H, CH), 6.45 (s, 2H, =CH), 6.93 (dd, *J* = 1.57; 8.19 Hz, 2H, ArH), 7.12 (s, 2H, ArH), 7.17 (d, *J* = 8.19 Hz, 2H, ArH), 7.34–7.35 (m, 2H, ArH), 7.41(dd, *J* = 8.19 Hz, 1H, ArH), 7.62–7.68 (m, 3H, ArH), 7.71–7.74 (m, 1H, ArH) ppm; UV (λ_max_, CH_2_Cl_2_**)**: 278 nm (*c* = 1.0 × 10^−4^, A = 1.00, ε = 1.0 × 10^4^).

*Polymer* (**17**). Light brown solid; yield = 75%; FTIR (ATR): υ = 3367, 3036, 2923, 2858, 1657, 1600, 1486, 1435, 1385, 1245, 1090, 796 cm^−1^; ^1^H-NMR (DMSO-*d*_6_, 500 MHz): δ = 1.27–1.32 (m, n6H, CH_3_), 1.34–1.37 (m, n6H, CH_3_), 1.96–1.98 (m, 4H, CH_2_), 2.13 (p, *J* = 6.30 Hz, 4H, CH_2_), 2.34 (s, 6H, CH_3_), 3.32–3.33 (m, n2H, CH), 3.10 (t, *J* = 6.30 Hz, 4H, CH_2_), 4.03–4.06 (m, 4H, CH_2_), 6.32 (s, 2H, =CH), 6.51 (s, 1H, CH), 6.94 (dd, *J* = 1.26; 8.19 Hz, 2H, ArH), 7.01 (s, 2H, ArH), 7.20 (d, *J* = 8.19 Hz, 2H, ArH), 7.72 (d, *J* = 7.88 Hz, 1H, ArH), 7.79 (d, *J* = 8.82 Hz, 2H, ArH), 7.95–7.97 (m, 3H, ArH), 8.06–8.10 (m, 2H, ArH); 8.36 (d, *J* = 9.46 Hz, 1H, ArH) ppm; UV (λ_max_, CH_2_Cl_2_**)**: 349 nm (*c* = 0.2 × 10^−3^, A = 2.45, ε = 1.2 × 10^4^).

*Polymer* (**18**). Light brown solid; yield = 70%; FTIR (ATR): υ = 3305, 3019, 2918, 2857, 1662, 1618, 1485, 1432, 1364, 1167, 1064, 766 cm^−1^; ^1^H-NMR (DMSO-*d*_6_, 500 MHz): δ = 1.28–1.31 (m, n6H, CH_3_), 1.36–1.39 (m, n6H, CH_3_), 1.94–1.97 (m, 4H, CH_2_), 2.12–2.16 (m, 4H, CH_2_), 2.25 (s, 6H, CH_3_), 3.04–3.09 (m, 4H, CH_2_), 3.28–3.31 (m, n2H, CH), 4.04–4.07 (m, 4H, CH_2_), 6.28 (s, 1H, CH), 6.40 (s, 2H, =CH), 6.49 (d, *J* = 8.19 Hz, 2H, ArH), 6.94–6.98 (m, 2H, ArH), 7.12 (d, *J* = 8.19 Hz, 1H, ArH), 7.16 (s, 2H, ArH), 7.18–7.20 (m, 2H, ArH), 7.37–7.43 (m, 2H, ArH), 7.49–7.52 (m, 2H, ArH), 8.11 (t, *J* = 8.19 Hz, 1H, ArH), 8.61 (d, *J* = 8.19 Hz, 1H, ArH) ppm; UV (λ_max_, CH_2_Cl_2_**)**: 300 nm (*c* = 0.5 × 10^−4^, A = 0.50, ε = 1.0 × 10^4^).

*Polymer* (**19**). Light brown solid; yield = 60%; FTIR (ATR): υ = 3386, 3019, 2929, 1622, 1615, 1488, 1437, 1388, 1180, 1098, 776 cm^−1^; ^1^H-NMR (DMSO-*d*_6_, 500 MHz): δ = 1.25–1.29 (m, n6H, CH_3_), 1.33–1.38 (m, n6H, CH_3_), 1.94–1.97 (m, 4H, CH_2_), 2.19 (p, *J* = 6.30 Hz, 4H, CH_2_), 2.26 (s, 6H, CH_3_), 3.17 (t, *J* = 6.30 Hz, 4H, CH_2_), 3.22–3.27 (m, n2H, CH), 3.73 (s, 2H, CH_2_), 4.11–4.15 (m, 4H, CH_2_), 6.43 (s, 1H, CH), 6.48 (s, 2H, =CH), 6.94 (d, *J* = 8.19 Hz, 2H, ArH), 7.12 (d, *J* = 3.48 Hz, 2H, ArH), 7.17 (d, *J* = 8.19 Hz, 2H, ArH), 7.26–7.29 (m, 2H, ArH), 7.41 (s, 1H, ArH), 7.43 (d, *J* = 7.56 Hz, 1H, ArH), 7.62 (dd, *J* = 1.57; 7.56 Hz, 1H, ArH), 7.67 (d, *J* = 7.56 Hz, 1H, ArH), 7.84 (s, 1H, ArH) ppm; UV (λ_max_, CH_2_Cl_2_**)**: 308 nm (*c* = 0.5 × 10^−4^, A = 1.22, ε = 2.4 × 10^4^).

## 4. Conclusions

In conclusion, bis(indolyl)methanes, which are found in cruciferous plants and marine sources, have shown different pharmaceutical activities such as anticancer, antiviral and antimicrobial properties. In this study, these compounds have been used for the first time in the textile industry as UV absorbers. They increased the UV protection factor of undyed fabric from +27 to +40–50. This shows that they are quite active compounds as UV absorbers. Furthermore, all printed fabrics were washed 25 times according to standard procedure and the results indicated that the novel compounds also carry antibacterial properties.

## Figures and Tables

**Table 1:** Properties of new compounds.

Product	Time (h)	Yield (%)	Mp (°C)	λ_max_/nm (Absorbance)
**2**	3	70	oil	-
**3**	2	55	141–142	315 (1.52)
**4**	2	67	118–119	310 (1.42)
**5**	2	60	102	300 (1.77)
**6**	3	52	-	315 (0.60)
**7**	2	52	-	310 (1.90)
**8**	2	47	-	315 (1.63)
**9**	2	75	oil	-
**10**	2	61	127–130	290 (1.79)
**11**	2	65	101–103	310 (1.77)
**12**	4	72	133–135	346 (1.60)
**13**	4	75	168–171	296 (1.47)
**14**	4	60	193–196	307 (1.50)
**15**	4	65	-	283 (1.20)
**16**	4	60	-	278 (1.00)
**17**	4	75	-	349 (2.45)
**18**	4	70	-	300 (0.50)
**19**	4	60	-	308 (1.22)

**Table 2:** GPC results of the polymers **6**–**8**, **15**–**19**.

Polymer	M_w_ (×10^5^ g/mol)	M_n_ (×10^5^ g/mol)
**6**	5.034	4.908
**7**	5.089	4.635
**8**	5.042	4.712
**15**	15.35	15.14
**16**	11.01	10.99
**17**	10.82	7.684
**18**	8.314	7.627
**19**	5.065	4.950

**Table 3:** UV protective features of absorbers.

UV Absorber Printed Fabric	UVA Transmittance * (315–400 nm)	UVB Transmittance * (280–315 nm)	UPF	UVA Blocking (%)	UVB Blocking (%)	UPF Label
reference	5.65	2.02	33.37	94.35	97.98	27
**6**	0.30	0.10	1300.1	99.70	99.90	40+
**7**	2.15	0.22	216.77	97.85	99.78	40+
**8**	3.22	0.38	146.12	96.79	99.62	40+
**15**	2.15	0.12	300.90	97.85	99.88	40+
**16**	1.47	0.10	381.05	98.53	99.90	40+
**17**	0.80	0.05	1314.43	99.20	99.95	40+
**18**	1.52	0.05	594.00	98.48	99.95	40+
**19**	0.38	49.98	1604.18	99.62	99.98	40+

* average.

**Table 4:** Antibacterial features of absorbers.

Fabric Printed	Washes	Bacteria Growth	Inhibition Zone (mm) *
reference	-	+	
**6**	25	-	0
**7**	25	-	0
**8**	25	-	0
**15**	25	-	0
**16**	25	-	0
**17**	25	-	0
**18**	25	-	0
**19**	25	-	0

* width of clear zone of inhibition. (positive = There is bacterial growth; negative = There is not bacterial growth).
